# The effect of music type in ketamine-assisted group therapy on treatment-resistant mental health conditions: a prospective observational study

**DOI:** 10.1192/j.eurpsy.2024.1480

**Published:** 2024-08-27

**Authors:** K. E. Sattler, T. Walia, V. Tsang

**Affiliations:** ^1^University of British Columbia, Vancouver; ^2^McMaster University, Hamilton, Canada

## Abstract

**Introduction:**

Currently, Ketamine is the only safe, effective, and widely used psychedelic-like medicine in Canada. It has demonstrated notably efficacy in providing relief to those experiencing treatment resistant mental health conditions. Pairing Ketamine treatment with psychotherapy, known as Ketamine Assisted Therapy (KAT), has been shown to yield more enduring outcomes. Work by Greenway et al. has demonstrated that playing music following ketamine administration for patients with bipolar disorder can help the patient feel more in control and reduce discomfort (*Greenway et al.* International Clinical Psychopharmacology 2021; 36 218-220).

**Objectives:**

The primary objective is to evaluate and compare the subjective clinical efficacy of two different types of music during ketamine-assisted group therapy. This will be explored through various validated psychiatric questionnaires, including the PHQ-9, GAD-7, and PCL-5. The secondary objective is to compare the objective changes in brain activity between the two music types. This will be evaluated using EEG data collected from MUSE headband before and after each ketamine-assisted therapy session.

**Methods:**

This study is a crossover trial of 32 participants undergoing ketamine-assisted therapy for treatment-resistant mental health conditions. Half of participants will undergo a KAT session with a “weightless” music playlist followed by a session with a “grounding” music playlist. The other half will do the same, in reverse order. All participants will complete several psychiatric questionnaires within 7 days of each session over email. Before and after each session, participants will play a simple game to test executive function while wearing a headband to measure EEG activity.

**Results:**

The absolute and relative changes to the scores of the questionnaires will be examined between participants and music conditions. The change in brain activity from pre-session to post-session will be compared between the different music conditions as well. As this is a crossover trial, any changes in outcomes due to order effects will be controlled for. Relevant demographic and medical factors will also be controlled for.

**Conclusions:**

To date, no studies have explored the influence that different types of music have on patients experience with KAT in a group therapy setting. With the results of this study, we hope to fine tune and improve the use of music in future KAT administration.

**Disclosure of Interest:**

None Declared

